# Zeno and Anti-Zeno Effects in Nonadiabatic Molecular Dynamics

**DOI:** 10.1021/acs.jpclett.3c01831

**Published:** 2023-08-09

**Authors:** Shriya Gumber, Oleg V. Prezhdo

**Affiliations:** † Department of Chemistry, University of Southern California, Los Angeles, California 90089, United States; ‡ Department of Physics and Astronomy, University of Southern California, Los Angeles, California 90089, United States

## Abstract

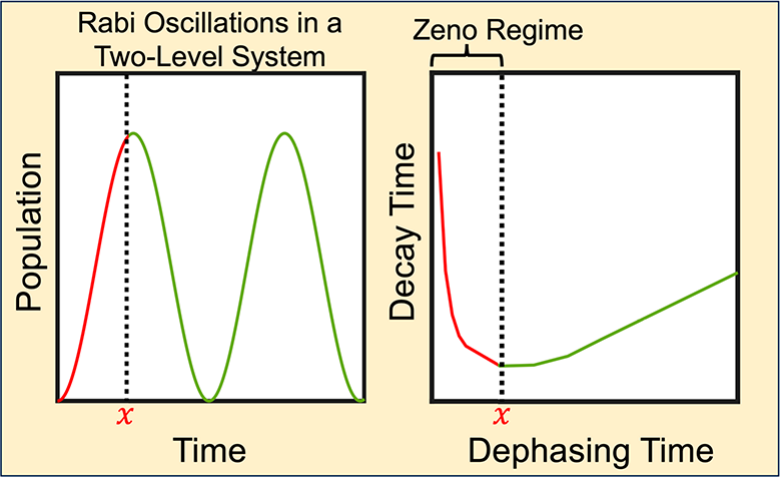

Decoherence plays an important role in nonadiabatic (NA) molecular dynamics (MD) simulations because it provides a physical mechanism for trajectory hopping and can alter transition rates by orders of magnitude. Generally, decoherence effects slow quantum transitions, as exemplified by the quantum Zeno effect: in the limit of infinitely fast decoherence, the transitions stop. If the measurements are not sufficiently frequent, an opposite quantum anti-Zeno effect occurs, in which the transitions are accelerated with faster decoherence. Using two common NA-MD approaches, fewest switches surface hopping and decoherence-induced surface hopping, combined with analytic examination, we demonstrate that including decoherence into NA-MD slows down NA transitions; however, many realistic systems operate in the anti-Zeno regime. Therefore, it is important that NA-MD methods describe both Zeno and anti-Zeno effects. Numerical simulations of charge trapping and relaxation in graphitic carbon nitride suggest that time-dependent NA Hamiltonians encountered in realistic systems produce robust results with respect to errors in the decoherence time, a favorable feature for NA-MD simulations.

Quantum nonadiabatic (NA) processes, such as charge and energy transfer, exciton formation, relaxation and recombination, bond dissociation, and other photochemical reactions, are frequently encountered in nature and technology.^1−11^ The most ambitious approach to model such processes is to treat the entire system quantum mechanically. However, due to its complexity and exponential computational cost, approximate semiclassical treatments cannot be avoided. Most NA simulations are performed in a mixed quantum-classical fashion, in which the electrons are treated quantum mechanically and nuclear motion is described classically or semiclassically. Fewest switch surface hopping (FSSH) has been the most popular algorithm for NA dynamics since published by Tully in 1990 because of its simplicity, robustness, and accuracy.^12^ In FSSH, the classical particles propagate on a single potential energy surface using Newton’s second law of motion unless a NA transition (hop) to another accessible surface takes place. The transition probability is determined based on the solution of the time-dependent Schrodinger equation (TD-SE) for the electrons moving in the field of classical nuclei. Switching to another electronic state is accompanied by velocity rescaling along the direction of the NA coupling to conserve the total quantum-classical energy, and if the nuclear kinetic energy is insufficient to hop to a certain state, such hops are excluded. The velocity rescaling and hop rejection make FSSH satisfy the detailed balance condition between transitions upward and downward in energy, leading to thermodynamic equilibrium.^13^ In FSSH, an ensemble of trajectories is generated, and the fraction of trajectories at each state determines the population of that state at a given time. The original FSSH formulated in the Hilbert space was extended into the Liouville space that explicitly includes coherences^14^ and was generalized to include the superexchange and many particle processes in the global flux surface hopping (GFSH) method.^15,16^

One of the major shortcomings to the applicability of FSSH is the problem of overcoherence, highlighted by Rossky and co-workers.^17−19^ Inclusion of decoherence effects in NA molecular dynamics (MD) methods has been up for discussion and developments in the past few decades.^20−34^ In FSSH, each trajectory among the swarm of trajectories is independent, and due to stochastic modeling, the trajectories can evolve along different paths. Nuclei corresponding to different trajectories evolve on different energy surfaces eventually, and on each surface, they experience different forces. As a result, the nuclei drive apart; i.e., each trajectory begins at the same initial nuclear configuration and electronic state and ends up at different final nuclear configurations and electronic states. The dynamics corresponding to different trajectories diverge in both nuclear position and electronic wave function. In the fully quantum treatment, such divergence of nuclear wave packets associated with different electronic states is known as decoherence.^35,36^ The effect of the quantum nuclear bifurcation on the electronic subsystem is not described by FSSH or related quantum-classical methods. The electronic amplitudes remain unaware that the total nuclear wave packet is breaking apart because the nuclei are treated classically. Different trajectories have no information about when to lose the electronic phase relation (coherence) with each other and instead carry forward infinite memory of the past, leading to electronic overcoherence. The NA-MD methods should take decoherence into account to prevent unphysical wave function superpositions from arising.^37^

Decoherence occurs in open quantum systems when the system interacts with a quantum environment. The environment acts by dampening the NA effects and transition probabilities between the electronic states. The loss of quantum system information to the environment is irreversible. An electronic density matrix (σ), used to represent the quantum state of a physical system, makes it convenient to describe the decoherence process mathematically:
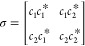
1Here, the diagonal elements represent the electronic state populations, and the off-diagonal elements include the overlap of nuclear wave packets correlated to states 1 and 2. Divergence of the nuclear wave packets causes decay of the off-diagonal elements of the reduced electronic density matrix. If the nuclei are treated classically, as in FSSH, the off-diagonal elements do not decay. Decoherence is particularly important when the nuclear wave packet moves through the region of strong coupling more than once.^38,39^ During the first crossing, a nuclear wave packet bifurcation takes place, with amplitudes assigned to the electronic states. Subsequently, the resultant wave packets diverge. At the second crossing, if the amplitudes are evolved without including decoherence effects, the calculated transition probabilities cause hopping to the wrong states, resulting in disastrous dynamics results. The transition rates calculated using NA-MD can be off by several orders of magnitude if decoherence is not included.^40,41^ NA-MD simulations are widely applied to study the reaction dynamics in molecular, condensed matter, and nanoscale systems that exhibit fast coherence loss.^42−49^ Hence, it is of the utmost importance that the NA-MD methods account for the decoherence effects. Many efforts have been made in the past to include the decoherence effects in electronic and vibrational excited state dynamics.^20−34^

Typically, NA-MD without decoherence overestimates the transition rates and probabilities. Inclusion of decoherence slows quantum dynamics, and rapid decoherence between energetically distant electronic states completely halts the population transfer. The extreme limit of decoherence, when population transfer stops, is called the quantum Zeno effect (QZE). QZE was demonstrated by Misra and Sudarshan, who studied the evolution of a quantum system, subject to frequent ideal measurements.^50^ In their results, an unstable molecule never decays to its stable ground state in the limit of infinitely frequent measurements. This is because, on every measurement, the wave function collapses to eigenstates of the measurement basis, in proportion to quantum populations of the states. If the system is in one of the eigenstates at time zero, it always collapses back to the initial state because the first derivative of the populations with respect to time is zero initially, as seen in the Rabi oscillation. The opposite was discovered later, indicating that if the measurements are not sufficiently rapid, then the transition can be accelerated by decoherence. This is called the quantum anti-Zeno effect (QAZE).^51−54^ Thus, decoherence can cause transitions to speed up or slow down. The decoherence time can vary due to changes in temperature, material composition, isotopic substitution, etc., providing means to tune the behavior of quantum systems.^55^ The study of crossover from the Zeno to anti-Zeno regime has been a subject of interest for quite some time. For instance, Chaudhry derived the general expression for the rate of decay of the initial quantum state subject to repeated measurements.^56^ The QZE and QAZE have been studied experimentally using various setups, including superconducting qubits, spin systems, etc.^57−66^ Decoherence carries importance for understanding quantum mechanical fundamentals, and with the increasing sophisticated quantum technologies, it plays key roles in open quantum systems, quantum computation, quantum information processing, quantum sensing, and other applications.^67−71^

It is important to have NA-MD methods that describe both QZE and QAZE, as these effects have been identified and studied experimentally. The Lindblad theory of open quantum systems provides a general framework for modeling quantum systems coupled to quantum baths.^72^ It can be formulated at both the ensemble averaged and individual trajectory levels.^73−76^ Particularly relevant for NA-MD simulations, the individual trajectory formulation of the theory of open quantum systems results in stochastic Schrodinger equations, which can be continuous but not differentiable or piecewise continuous and differentiable, describing quantum diffusion or quantum jump processes.^73−76^ These two types of quantum trajectory descriptions have led respectively to the stochastic mean-field^20^ (SMF) and decoherence induced surface hopping^27^ (DISH) approaches to NA-MD. Because the decoherence process provides the physical basis for trajectory branching,^20,77^ hops in SMF and DISH happen at decoherence events, and there is no need for additional ad hoc rules, as in FSSH^12,14^ and GFSH.^15,16^ Solution of the TD-SE is used in FSSH as an auxiliary step needed to calculate time and configuration dependent transition rates between states,^78^ and the observables are obtained from trajectory statistics rather than the TD-SE. In contrast, the observables in the SMF and DISH methods are obtained directly from the stochastic TD-SE. The DISH method is widely used to model excited state dynamics in a broad range of systems.^79−85^

In this Letter, we demonstrate that both QZE and QAZE are common in NA-MD simulations of molecular, condensed phase, and nanoscale systems. Generally, decoherence slows NA transitions when they occur across energy gaps of tens of electronvolts or more. However, in many cases, systems operate in the anti-Zeno regime. Hence, it is important for NA-MD approaches to describe both QZE and QAZE. The QZE is always observed for a sufficiently short decoherence time. As the coherence time grows, a system switches to either anti-Zeno or random behavior. Numerical simulations performed with graphitic carbon nitride (g-C_3_N_4_) suggest that realistic systems with fluctuating time-dependent Hamiltonians are more likely to show the anti-Zeno behavior because the randomness tends to average out. This is a favorable feature for NA-MD simulations, making them robust to errors in the decoherence time and other system parameters. The coherence time can be tuned experimentally, e.g., by disorder or temperature, allowing one to operate realistic systems in both the QZE and QAZE regimes.

To perform numerical simulations, we use two-dimensional g-C_3_N_4_ (Figure 1), which is a popular metal-free semiconductor extensively studied for applications in photo- and electrocatalysts, photovoltaics, biosensing, etc.^86−88^ Structural defects are introduced into g-C_3_N_4_ to improve its performance. We consider g-C_3_N_4_ containing oxygen and nitrogen defects (ON-gcn), which are introduced to optimize both its work function (electrode potential) and charge separation. As highlighted by the dashed green circles in Figure 1a, ON-gcn includes (i) an N-vacancy defect, which leads to the formation of a C–C bond, (ii) CN, NH, and NH_2_ groups arising from breaking of two C–N bonds, and (iii) O-doping, where O replaces an N atom in a tri-*s*-triazine unit. The system includes both delocalized band states and localized defect states, with a range of energy gaps between them, allowing us to consider charge trapping and recombination on picosecond and nanosecond time scales. While delocalized states form bands that are described with multiple *k*-points, the conduction band minimum (CBM) and valence band maximum (VBM) of g-C_3_N_4_ correspond to the Γ *k*-point. Because localized defect states do not depend on the *k*-point, only the Γ *k*-point can be used in the present system.^79,80^ Focusing on a set of two-level systems stemming from ON-gcn, we model quantum dynamics in the original material by applying the DISH and FSSH approaches to the ab initio NA Hamiltonian. We also vary the Hamiltonian parameters systematically to demonstrate the QZE and QAZE regimes. The details of the ab initio DISH and FSSH calculations on ON-gcn are described elsewhere.^79,80^

**Figure 1:**
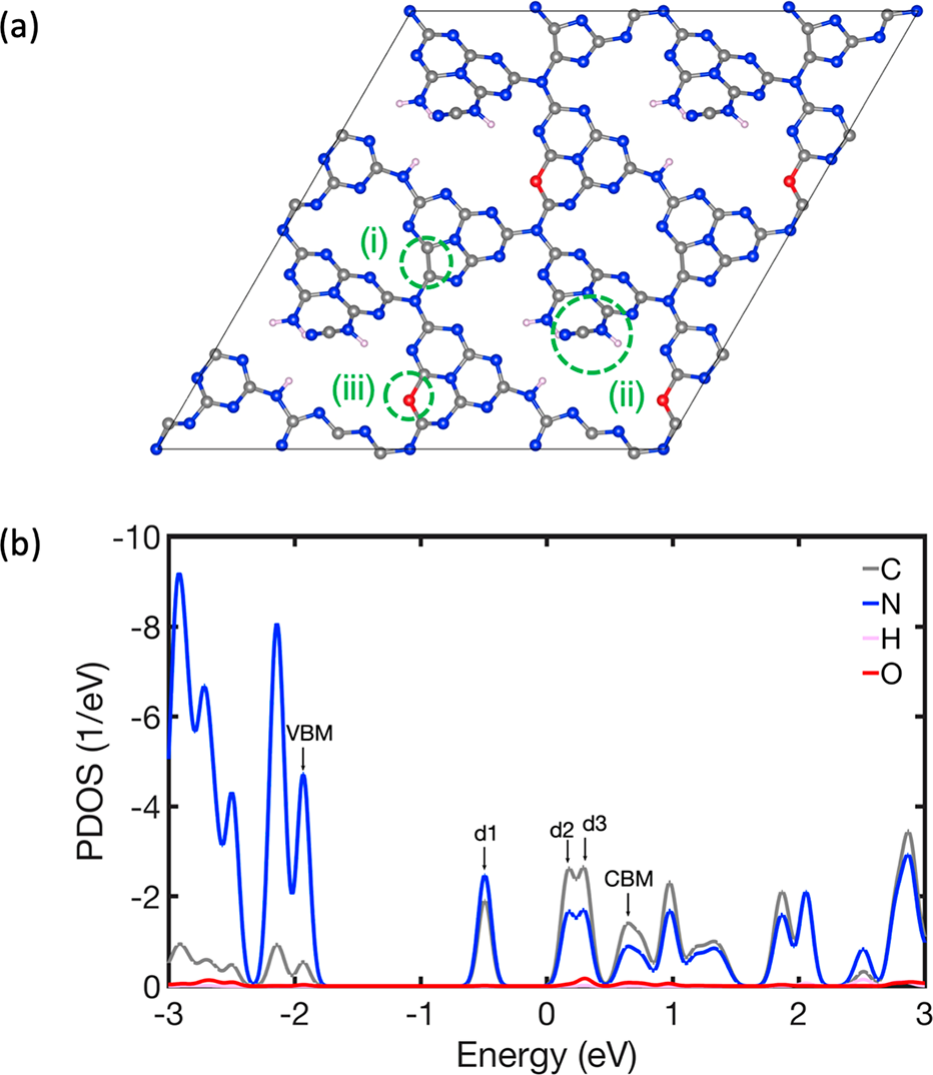
(a) Optimized geometry of the dual-defect-modified graphitic carbon nitride (ON-gcn), containing two N defects: (i) N vacancy, which results in the formation of a C–C bond, (ii) CN defect, which gives the NH and NH_2_ groups, and (iii) an O defect, formed by doping N of a tri-*s*-triazine unit with an O atom. Color scheme: C, gray; N, blue; O, red; H, pink. (b) Projected density of states (PDOS) of the spin-down component of the ON-gcn system, separated into contributions from C, N, O, and H. There are three midgap defect states, marked as d1, d2, and d3, between the valence band maximum (VBM) and the conduction band minimum (CBM).

The decoherence times are evaluated as the pure-dephasing times of the optical response theory (Table 1).^89,90^ The pure-dephasing function is obtained using the second-order cumulant approximation
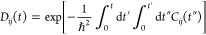
2Here, *C*_*ij*_(*t*) is the autocorrelation function
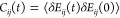
3of nuclear-driven fluctuations of the electronic energy gap from its average value
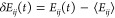
4

The projected density of states (PDOS) of ON-gcn is shown in Figure 1b. In the simulations, we consider the VBM, the CBM, and the defect states d1, d2, and d3. The d1 state is occupied and is a hole trap, while the d2 and d3 states are empty and are electron traps.^79^ We consider transitions between the vbm–d1, d1–d2, and d3–cbm state pairs. These state pairs represent a range of energy gaps, 1.5, 0.7, and 0.4 eV, respectively, typical of many modern systems. Simplifying the complex ON-gcn system into a set of two-level systems allows us to perform a straightforward analysis of the QZE and QAZE regimes.

The NA coupling (NAC) matrix elements between the states are computed as
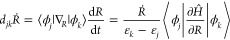
5The NAC values vary opposite to the energy gaps; i.e., small energy gap correlates with large coupling. On average, d3–cbm has an order of magnitude higher NAC than vbm–d1 and d1–d2 (Table 1). A significant difference between the average absolute and RMS values of the NAC suggests that the d3–cbm energy gap has a large fluctuation, and hence, the transition probability fluctuates as well. Fluctuations of the VBM, d1, d2, d3, and CBM energies along a 5 ps trajectory are shown in Figure 2a, and the corresponding evolutions of the absolute NAC values are shown in Figure 2c. Shorter, 60 fs trajectories are shown in Figures 2b,d, allowing one to observe typical oscillations periods. Transitions across larger and smaller energy gaps represent different physical processes. For example, in charge-carrier dynamics in semiconductors, charge trapping occurs across smaller gaps, while charge recombination takes place across larger energy gaps.

**Figure 2:**
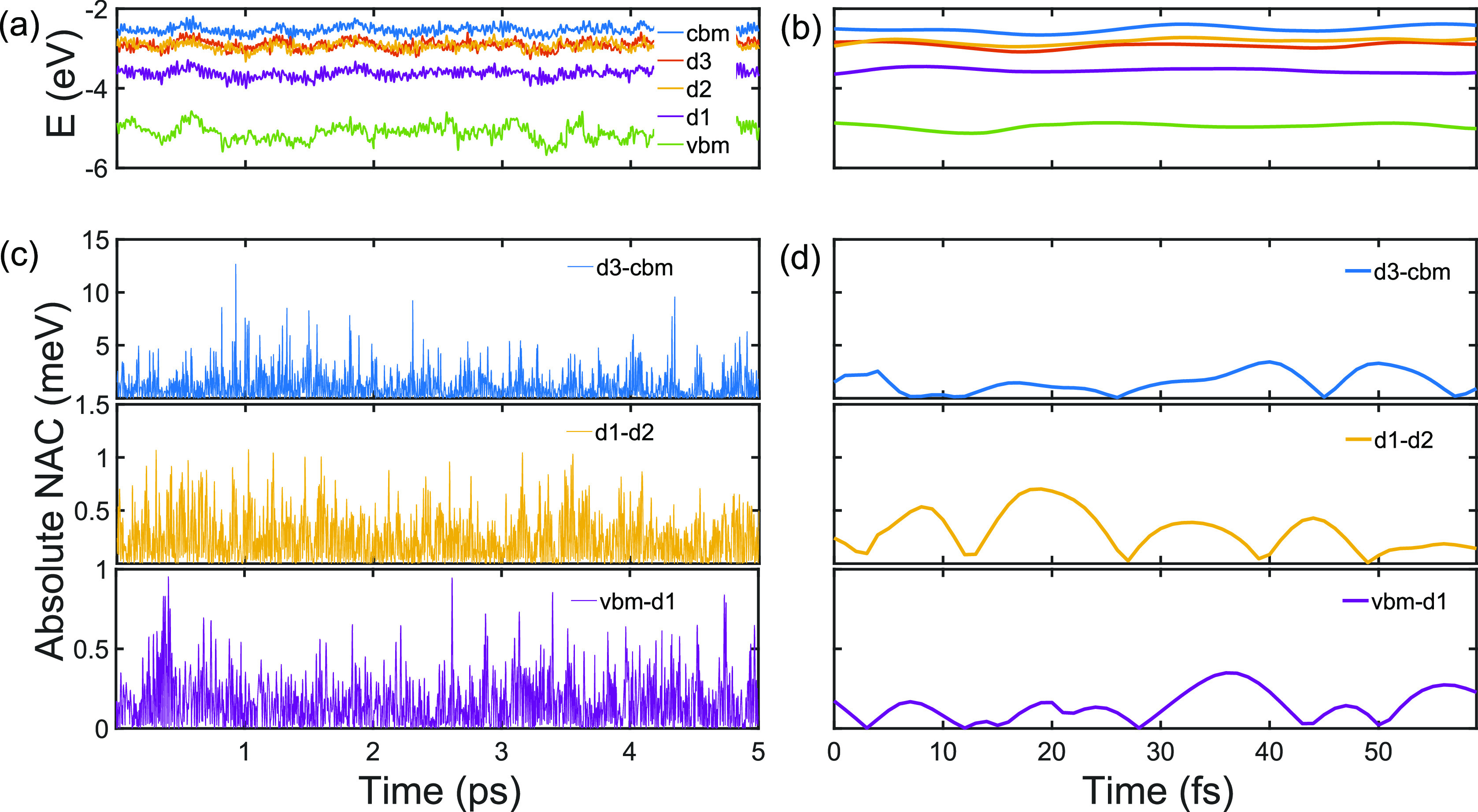
Energies of the adiabatic electronic states in the ON-gcn system (Figure 1) obtained every 1 fs time step from an MD trajectory (a) for 5 ps and (b) zoomed to 60 fs to display fluctuations. The absolute value of the nonadiabatic coupling (NAC) between the vbm–d1, d1–d2, and d3–cbm adjacent electronic state pairs (c) calculated along the 5 ps trajectory and (d) zoomed to display NAC for 60 fs. The corresponding average values of the energy gaps and the average absolute and root-mean-square values of the NAC are provided in Table 1.

**Table 1:** Average Energy Gap, Average Absolute and Root Mean-Square (RMS) Nonadiabatic Coupling (NAC), and Pure-Dephasing Time for the Three Two-Level Systems^a^

	av energy gap (eV)	av absolute NAC (meV)	RMS NAC (meV)	pure-dephasing time (fs)	freq, Ω (1/fs)	period, τ = 2π/Ω (fs)
vbm–d1	1.5	2.4	3.1	6.0	2.3	2.8
d1–d2	0.7	3.5	4.4	9.6	1.1	6.0
d3–cbm	0.4	16.3	23.1	9.8	0.6	10.9

a The time-dependent energies and NACs are shown in Figure 2. The pure-dephasing times are computed using the second-order cumulant approximation of the optical response theory (eq 2). The frequency and period are calculated using the Rabi formula, eqs 6 and 8. These values match the numerical simulations (Figure 3a–c).

We performed several sets of NA-MD simulations. First, we compare the FSSH and DISH methods for the true ab initio NA Hamiltonian of the ON-gcn system. The comparison highlights the strong influence that decoherence has on quantum transitions in NA-MD. We consider two alternative FSSH implementations. In one case, we follow the conventional FSSH algorithm in which the TD-SE is propagated continuously. In the other case, we reset the TD-SE after every stochastic hop, mimicking the decoherence process in which the decoherence time scale (elastic scattering, pure dephasing) coincides with the time scale of the transitions (inelastic scattering, dissipation). Second, we compare DISH calculations performed with the true ab initio NA Hamiltonian and the NA Hamiltonian, in which the time-dependent energy gaps and NAC are replaced with their average values (Table 1). Such a comparison highlights the influence of fluctuations on NA-MD results. Third, we systematically vary the decoherence time in the DISH simulations with both time-dependent and time-independent NA Hamiltonians to observe the QZE and QAZE and to establish in which regime ON-gcn and other systems tend to operate.

A comparison between the FSSH and DISH methods is presented in Figure S1. In all cases, DISH exhibits much slower dynamics than FSSH. The cases with a larger energy gap, corresponding to a smaller NAC and shorter pure-dephasing times (Table 1), exhibit larger differences between FSSH and DISH. This result confirms that decoherence slows quantum dynamics and that decoherence effects are most important for slow transitions across large energy gaps. Relaxation across dense manifold of states can be well described by FSSH, ignoring decoherence effects.^82,91,92^ One can model decoherence in FSSH by collapsing the wave function to the newly occupied state after each hop. Such a method assumes that the pure-dephasing and relaxation time scales are the same; however, in general, there is no physical basis for such an assumption. The results show that for transitions across large energy gaps the pure-dephasing time is much faster than the relaxation time, and including decoherence effects into FSSH using this simple way makes little difference; compare FSSH-1 and FSSH-2 in Figure S1a,b.

Next, we compare quantum dynamics for the time-dependent and constant NA Hamiltonians. The parameters of the constant Hamiltonian are taken to be the averages of the time-dependent Hamiltonian (Table 1). Solutions of TD-SE shown in Figure 3 demonstrate that fluctuations of the Hamiltonian parameters induce fluctuations in the quantum dynamics. While the frequencies observed in the quantum dynamics remain largely the same, the amplitudes of the population oscillation can vary by more than an order of magnitude between the cases with the time-dependent and time-independent Hamiltonians. Such a difference in the behavior of the solution of the TD-SE can have a strong effect on both FSSH and DISH simulations because the hopping probability increases significantly when the quantum state population changes fast in FSSH or reaches large values in DISH. Note that the fluctuations in the NAC are of the same frequency or somewhat slower than the quantum dynamics frequencies; compare Figures 2d and 3.

**Figure 3:**
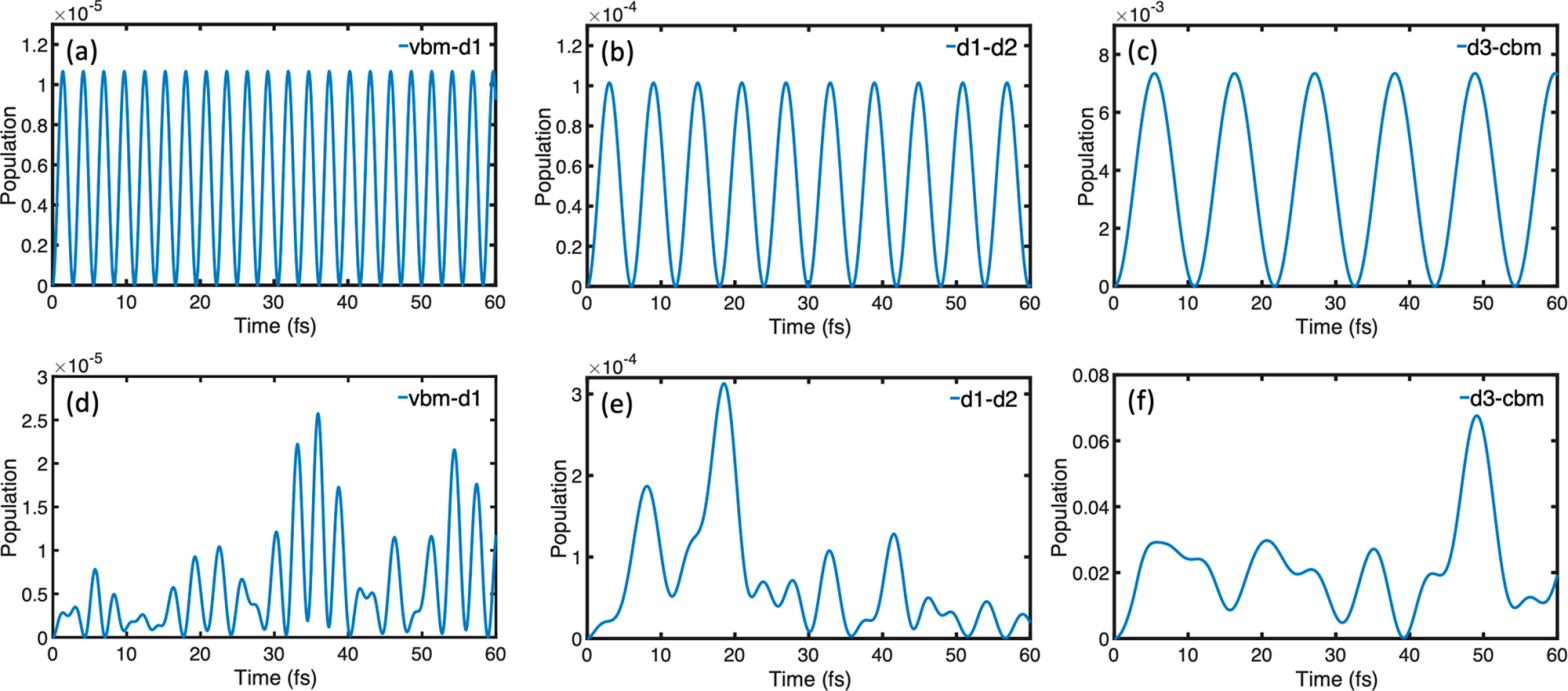
Evolution of the ground state population in the three two-level systems: vbm–d1, d1–d2, and d3–cbm, obtained by solving the time-dependent Schrödinger equation for (a–c) the time-independent Hamiltonians, as listed in Table 1, and (d–f) the time-dependent Hamiltonians calculated with a 1 fs time step along the 5 ps trajectory for the ON-gcn system (Figure 1). The population oscillates in (a–c) in accordance with the Rabi frequency (eq 6). The oscillation is less regular in (d–f) because the energy gap and NAC vary in time on a time scale similar to the population (Figure 2).

A constant two-state Hamiltonian allows a straightforward analytic analysis, as detailed in the Supporting Information. The state populations oscillate with the Rabi frequency
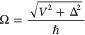
6where  is half the energy gap between the two states and *V* is the time-independent coupling. It can be inferred from this formula that the oscillations are more frequent for a two-level system, with states energetically farther from each other. Confirmed by Figure 3, this fact has a direct consequence for the QZE because QZE occurs when the decoherence time is faster than the initial stage of the Rabi oscillation. The amplitude of the oscillation is given by
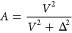
7This formula demonstrates that only a small fraction of the population oscillates between the states when the energy difference is large and the coupling is small, as confirmed by Figure 3. The period of the Rabi oscillation is given by the inverse of its frequency:
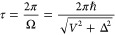
8The Rabi oscillation period helps in identifying the decoherence time for crossover from the Zeno regime to the anti-Zeno regime.

The DISH algorithm is used to model the NA dynamics in the three two-level systems arising from the ON-gcn material. Both time-dependent and time-independent NA Hamiltonians are considered. The NA-MD data for all simulations are shown in Figures S2 and S3. The relaxation times obtained by exponential fitting the NA-MD data are shown in Figure 4. The decoherence time is varied from 0.2 to 30 fs. The quantum dynamics is the fastest for the d3–cbm system with the smallest energy gap and the largest NAC, while it is the slowest for the vbm–d1 system with the largest energy gap and the smallest NAC (Table 1). Comparing the time-independent and time-dependent Hamiltonians, top and bottom panels in Figure 4, we observe that fluctuations in the Hamiltonian parameters make the dynamics faster. This is because the Hamiltonian fluctuations create fluctuations in the quantum state populations (Figure 3), and large values of state populations increase the hopping probability. We also observe that fluctuations in the NA Hamiltonian make the dependence of the population decay time on the decoherence time more uniform. The systems with the true NA Hamiltonian that fluctuates in time exhibit a clear transition from the Zeno to the anti-Zeno regime (Figures 4d–f), while the model systems with the constant Hamiltonian exhibit more random behavior above the Zeno to anti-Zeno crossover time (Figures 4a–c). The dependence of the quantum dynamics on the coherence time above the crossover time is a complicated function of the properties of the environment and the system–bath coupling, as analyzed in refs (56 and 61). The more uniform dependence of the population decay time on the decoherence time seen with the true fluctuating Hamiltonians allows for a robust modeling of realistic materials because the modeling is not sensitive to errors arising from the ab initio calculations of the NA Hamiltonian and DISH statistics.

**Figure 4:**
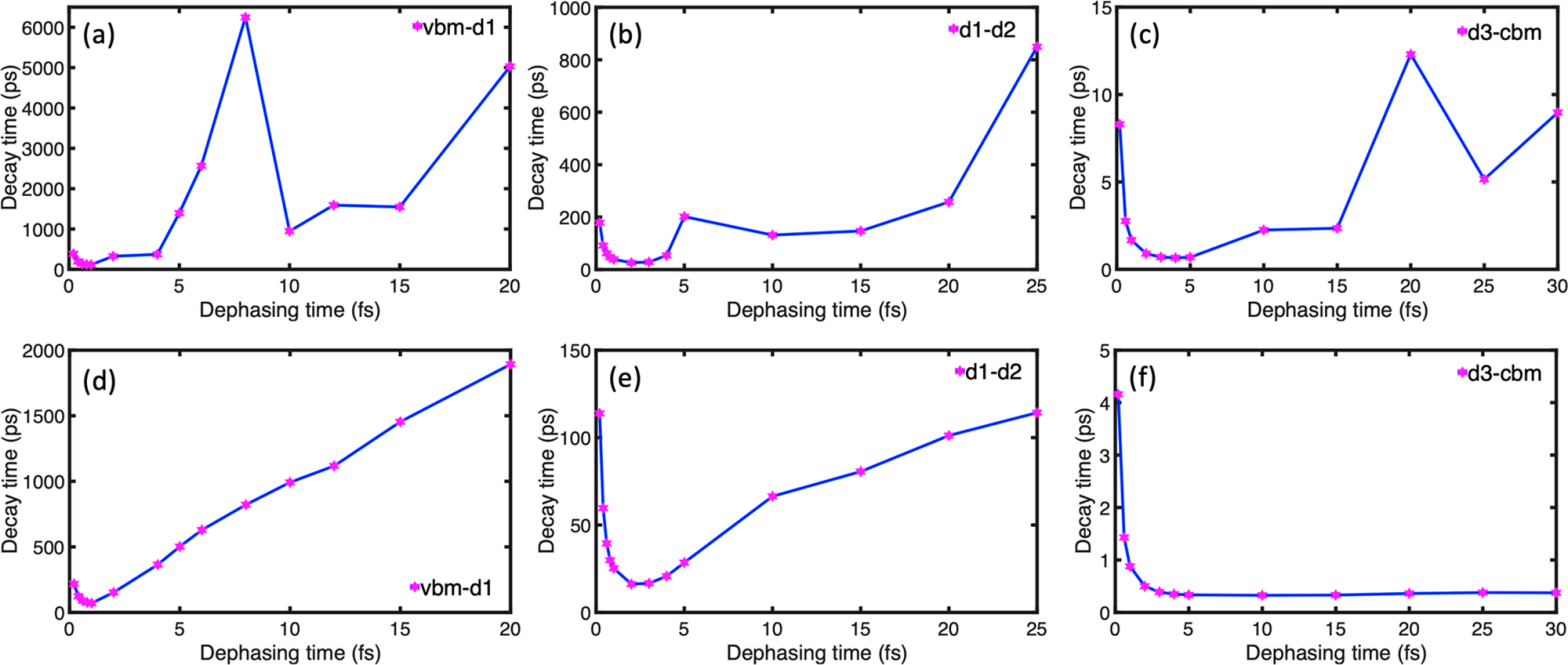
Population decay time as a function of the pure-dephasing/decoherence time in the vbm–d1, d1–d2, and d3–cbm two-level systems. For (a), (b), and (c), the energy gap and coupling are constant and equal to the average gap and average absolute nonadiabatic coupling (Table 1). For (d), (e), and (f), the Hamiltonians are time-dependent, corresponding to Figure 2. The population decay data used to derive the decay times are shown in Figures S2 and S3. In all cases, the decay time increases with decreasing pure-dephasing time in the initial part of the plot, showcasing the quantum Zeno effect. As the pure-dephasing time increases, there is a crossover from the Zeno to anti-Zeno or random behavior. The crossover times are 5 fs or less, while the actual pure-dephasing times calculated using eq 2 are 6.0, 9.6, and 9.8 fs (Table 1), indicating that ON-gcn operates in the anti-Zeno regime. This is typical of many real systems.

The QZE relies on collapse of the evolving wave function onto the initial state, induced by a measurement. In NA-MD simulations, such a measurement is performed on the electronic subsystem by the nuclear bath. In the absence of a measurement, the electronic subsystem undergoes a Rabi oscillation (Figure 3). The QZE occurs if the measurement is performed before the initial wave function has time to develop a superposition with other states. Therefore, the decoherence time, which defines the time of the measurement, should be a fraction of the Rabi oscillation period. Because the Rabi oscillation period is inversely proportional to the energy gap (eq 8), systems with larger energy gaps traverse from the Zeno to the anti-Zeno regime at shorter decoherence times. This is confirmed by the DISH simulations in Figure 4.

Notably, even for the smallest energy gap of 0.4 eV considered here, the transition from the Zeno to the anti-Zeno regime occurs at a decoherence time of less than 5 fs (Figure 4). In comparison, the true decoherence times of the systems are 6 fs or larger (Table 1). Therefore, one concludes the anti-Zeno regime prevails in many, if not most, NA-MD simulations of transitions across significant energy gaps; i.e., a decrease of the decoherence time accelerates quantum dynamics. Experimentally, the decoherence time can be shortened by increasing the temperature or chemical disorder. At the same time, comparison of the FSSH and DISH results (Figure S1) demonstrates that decoherence effects slow down the dynamics compared to the fully coherent case. Hence, decoherence effects should be included in NA-MD simulations of transitions occurring across significant energy gaps. Whether a particular system is in the Zeno or anti-Zeno regime can be estimated by comparing the Rabi period (eq 8), with the decoherence time. The decoherence times evaluated for a broad range of systems are longer than 5 fs,^29,42−49^ suggesting that the anti-Zeno regime should be very common. The analysis reported here indicates that it is important for a NA-MD method to describe both the QZE and QAZE.

In conclusion, we have demonstrated that decoherence effects have a strong influence on quantum transitions in NA-MD simulations, when such transitions occur across relatively large energy gaps of several tens of electronvolts or more. Depending on the decoherence time, the systems can be in the quantum Zeno or anti-Zeno/random regime; i.e., the transition time can both decrease and increase as a function of the decoherence time. To observe the QZE, the decoherence time should be several times shorter than the Rabi period. Hence, to achieve the QZE effect for a transition across a 0.1 eV energy gap, decoherence should be on the order of 10 fs or faster, and for a transition across a 1 eV gap, decoherence should be on the order of 1 fs. In many cases, the decoherence times are longer than these, and the systems operate in the anti-Zeno regime. The QZE always occurs for sufficiently short coherence times. As the coherence time increases, the system can exhibit both anti-Zeno and random behavior. The reported analysis suggests that realistic systems with fluctuating time-dependent Hamiltonians are more likely to show the anti-Zeno behavior because the randomness tends to average out. All three cases considered here, involving transitions in the defect doped g-C_3_N_4_ across energy gaps from 0.4 to 1.5 eV, demonstrate that the QAZE is very common and that the majority of materials operate in the anti-Zeno regime. Rooted in the theory of open quantum systems, the DISH approach to NA-MD captures both QZE and QAZE effects, making it suitable for NA-MD simulations of molecular, condensed phase, and nanoscale systems. Quantum coherence and decoherence effects play particularly important roles in rapidly developing quantum technologies, such as quantum computing, quantum information processing, quantum sensing, etc. Experimentally, the coherence time can be tuned by the temperature and structural disorder, allowing one to observe both QZE and QAZE in many realistic systems.

## Supporting Information Available

Comparison of FSSH and DISH results; DISH results for all systems; analytic examination of the 2-level system (PDF)
