# Supplementary material for: Zeno and Anti-Zeno Effects in Nonadiabatic Molecular Dynamics
Source: J Phys Chem Lett. 2023 Aug 9;14(32):7274–82. doi: 10.1021/acs.jpclett.3c01831 (PMC10440816; doi:10.1021/acs.jpclett.3c01831)
Supplement: Supplementary file 1 — jz3c01831_si_001.pdf [file jz3c01831_si_001.pdf]

Supplementary Information

Zeno and Anti-Zeno Effects in Nonadiabatic Molecular Dynamics

Shriya Gumber[1] and Oleg V. Prezhdo[1,2,*]

[1] Department of Chemistry, University of Southern California, Los Angeles, CA 90089, USA
[2] Department of Physics and Astronomy, University of Southern California, Los Angeles, CA 90089, USA

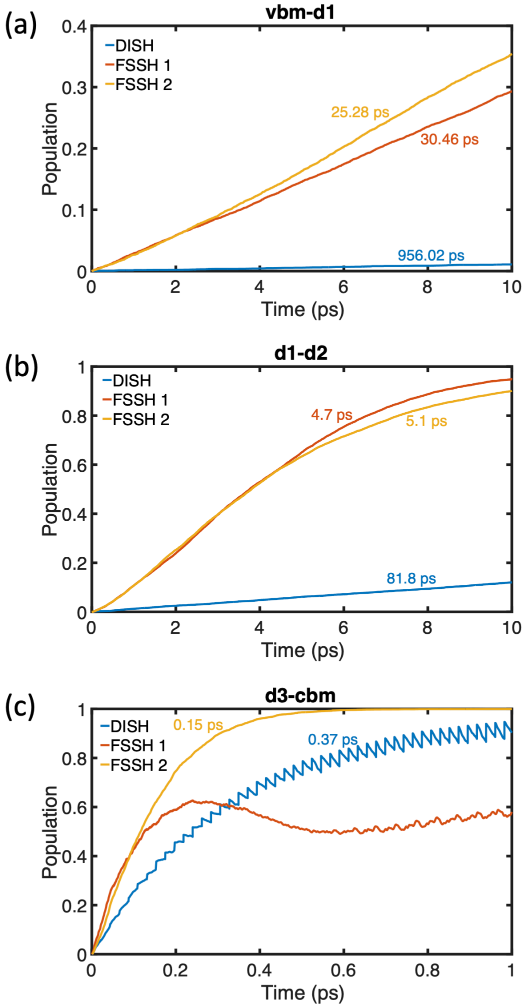

Figure S1. Comparison of the fewest switch surface hopping (FSSH) algorithm with the decoherence induced surface hopping (DISH) method for population growth of the lower energy state with the system prepared in the higher energy state for **(a)** vbm-d1, **(b)** d1-d2, **(c)** d3-cbm. The shown timescales are obtained by exponential fitting. In the DISH scheme, the time-dependent Schrodinger equation (TD-SE) is reset at every decoherence event. The decoherence time is calculated as the pure-dephasing time of the optical response theory based on the energy gap fluctuation. In the FSSH-1 scheme, the TD-SE is reset after every stochastic hop. The FSSH-2 follows the conventional FSSH algorithm in which the TD-SE is propagated continuously. The DISH dynamics is slower than both FSSH schemes, which show similar timescales in all three cases.

*prezhdo@usc.edu

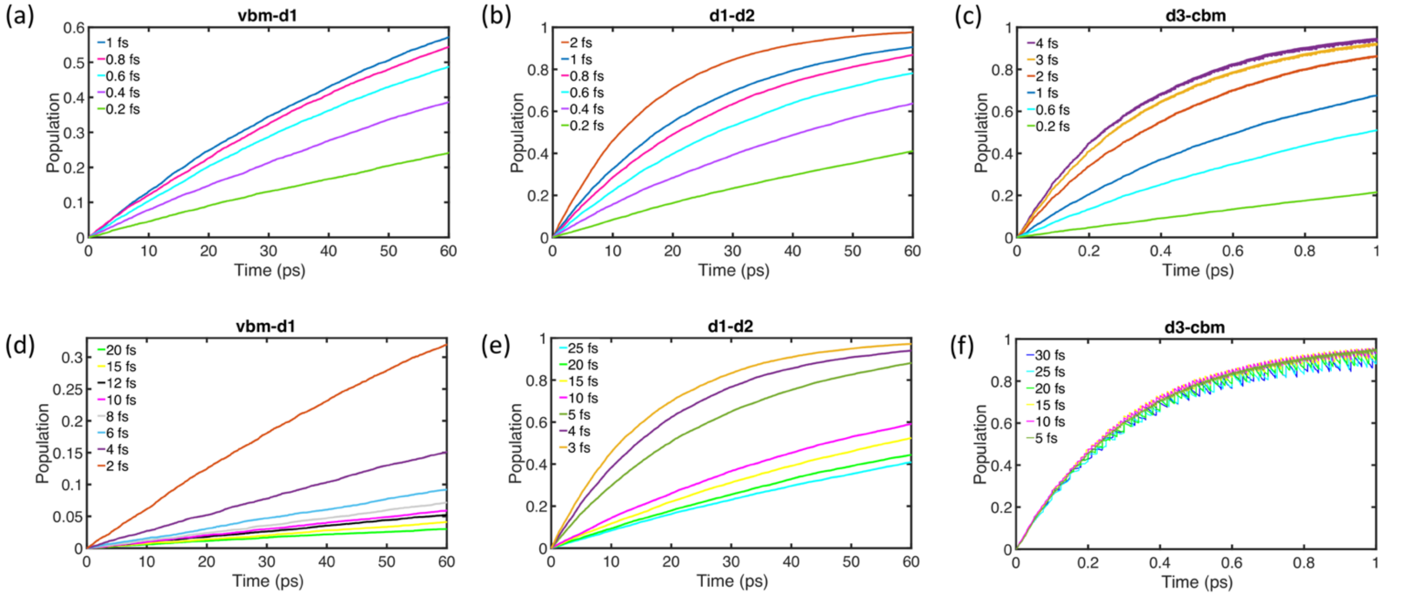

Figure S2. Evolution of the final state population in all three two-level systems under investigation (vbm-d1, d1-d2, d3-cbm). The energy gap and NAC are time-dependent, as obtained for the ON-gcn system shown in Figure 1. Each plot displays the dynamics of excited electron for different decoherence times as shown in the legends. The time constants obtained by exponential fits are plotted against the coherence time in Figure 5a-c. The upper panel **(a, b, c)** shows the Zeno regime: the dynamics are faster for longer coherence times. The lower panel **(d, e, f)** demonstrate the anti-Zeno and random behaviors: either the dynamics slow down for longer coherence time or show no correlation.

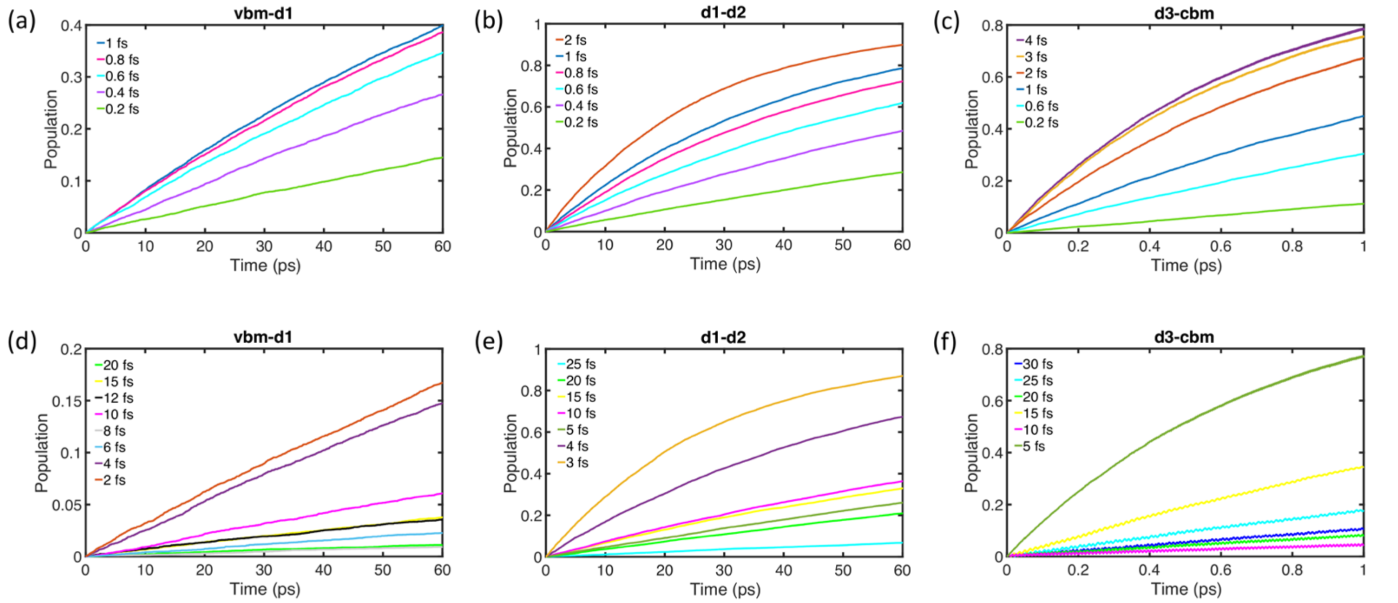

Figure S3. Same as Figure S1, but for the constant energy gap and NAC values as given in Table 1. Overall, the dynamics are slower for this case compared to the time-dependent case. The time-constants of exponential fits are plotted against the coherence time in Figure 5d-f.

General treatment of a two-level system

For a two-level system with eigenstates $|\varphi_a\rangle$ and $|\varphi_b\rangle$, respective energies E_a and E_b, and hamiltonian H_o, we have $H_o = \begin{bmatrix} E_a & 0 \\ 0 & E_b \end{bmatrix}$, such that,

$$H_o|\varphi_a\rangle = E_a|\varphi_a\rangle, \text{ and}$$

$$H_o|\varphi_b\rangle = E_b|\varphi_b\rangle$$

In particular, the two basis states $|\varphi_a\rangle$ and $|\varphi_b\rangle$ are considered to be orthonormal, i.e., $\langle\varphi_i|\varphi_j\rangle = \delta_{ij}$. In the presence of a time-independent coupling V, such as the nonadiabatic coupling, written as $\begin{bmatrix} 0 & V_{ab} \\ V_{ba} & 0 \end{bmatrix}$, the total Hamiltonian adds up to,

$$H = H_o + V = \begin{bmatrix} E_a & V_{ab} \\ V_{ba} & E_b \end{bmatrix}$$

Since V should be Hermitian, i.e., $V_{ab}^* = V_{ba}$, the total Hamiltonian can be re-written as:

$$H = \begin{bmatrix} E_a & Ve^{-i\varphi} \\ Ve^{i\varphi} & E_b \end{bmatrix}$$

To obtain the two eigen vectors, we solve the secular equation:

$$|H - \lambda I| = 0$$

Defining to variables $E = \frac{E_a+E_b}{2}$, and $\Delta = \frac{E_a-E_b}{2}$ we express the eigen energies as:

$$E_+ = E + \sqrt{\Delta^2 + V^2}$$

$$E_- = E - \sqrt{\Delta^2 + V^2}$$

corresponding to the eigenvectors written in terms of the original basis vectors $|\varphi_a\rangle$ and $|\varphi_b\rangle$,

$$|\psi_+\rangle = \cos\frac{\theta}{2}e^{-\frac{i\varphi}{2}}|\varphi_a\rangle + \sin\frac{\theta}{2}e^{\frac{i\varphi}{2}}|\varphi_b\rangle$$

$$|\psi_-\rangle = -\sin\frac{\theta}{2}e^{-\frac{i\varphi}{2}}|\varphi_a\rangle + \cos\frac{\theta}{2}e^{\frac{i\varphi}{2}}|\varphi_b\rangle$$

where

$$\tan\theta = \frac{V}{\Delta}$$

In the case of a strong coupling, $|V| \gg |\Delta|$

$$|\psi_\pm\rangle = \frac{1}{\sqrt{2}}(|\varphi_a\rangle \pm |\varphi_b\rangle)$$

The stationary eigenstates in the presence of a strong and time-constant coupling V, are symmetric and antisymmetric combinations of the basis states. Considering that $|\varphi_a\rangle$ and $|\varphi_b\rangle$ are a complete basis states for the two-level system, the wave function can be written as a linear combination of these states,

$$|\psi(t)\rangle = c_a(t)|\varphi_a\rangle + c_b(t)|\varphi_b\rangle$$

Here, $c_a(t)$ and $c_b(t)$ are time-dependent coefficients, such that their squares give the probabilities of the system being in the respective states $|\varphi_a\rangle$ and $|\varphi_b\rangle$. The time-dependent Schrodinger equation (TD-SE) for this system is defined as:

$$i\hbar \frac{d}{dt}|\psi(t)\rangle = H|\psi(t)\rangle$$

Representing this equation in the basis vectors, we have,

$$i\hbar \frac{d}{dt}c_a(t) = E_a c_a(t) + Ve^{i\varphi}c_b(t)$$

$$i\hbar \frac{d}{dt}c_b(t) = E_b c_b(t) + Ve^{-i\varphi}c_a(t)$$

Initially, at time $t = 0$, we consider that the system is in only one of the states, say $|\varphi_a\rangle$,

$$|\psi(0)\rangle = |\varphi_a\rangle$$

Using the stationary states $|\psi_+\rangle$ and $|\psi_-\rangle$ calculated above,

$$|\psi(0)\rangle = |\varphi_a\rangle = \cos\frac{\theta}{2}e^{\frac{i\varphi}{2}}|\psi_+\rangle - \sin\frac{\theta}{2}e^{\frac{i\varphi}{2}}|\psi_-\rangle$$

and the time-dependent part,

$$|\psi(t)\rangle = e^{-\frac{iE_+t}{\hbar}}\cos\frac{\theta}{2}e^{\frac{i\varphi}{2}}|\psi_+\rangle - e^{-\frac{iE_-t}{\hbar}}\sin\frac{\theta}{2}e^{\frac{i\varphi}{2}}|\psi_-\rangle$$

Probability amplitude of finding the system at time t in the state $|\varphi_b\rangle$ is:

$$\langle\varphi_b|\psi(t)\rangle = e^{\frac{i\varphi}{2}}(e^{-\frac{iE_+t}{\hbar}}\cos\frac{\theta}{2}\langle\varphi_b|\psi_+\rangle - e^{-\frac{iE_-t}{\hbar}}\sin\frac{\theta}{2}\langle\varphi_b|\psi_-\rangle)$$

$$= e^{\frac{i\varphi}{2}}\sin\frac{\theta}{2}\cos\frac{\theta}{2}(e^{-\frac{iE_+t}{\hbar}} - e^{-\frac{iE_-t}{\hbar}})$$

The probability is given by,

$$P = |\langle\varphi_b|\psi(t)\rangle|^2 = \sin^2\theta\sin^2\frac{E_+ - E_-}{2\hbar}t$$

On simplifying the equation,

$$P = \frac{V^2}{V^2 + \Delta^2}\sin^2\frac{\sqrt{V^2+\Delta^2}}{\hbar}t$$

Defining $\Omega = \frac{\sqrt{V^2+\Delta^2}}{\hbar}$, the Rabi frequency, we obtain

$$P_{ba} = \frac{V^2}{V^2+\Delta^2}\sin^2\Omega\, t$$

It is a triangular function; the probability oscillates between φ_a and φ_b. The period of oscillation is given by $\frac{2\pi}{\Omega}$.
